# Role of ^18^ F-FDG PET-CT in Urethral Malignant Melanoma and Analysis of the UK Guidelines on Ano-uro-genital Melanomas

**DOI:** 10.1055/s-0043-1771280

**Published:** 2023-09-06

**Authors:** Abhishek Mahato, Anurag Jain, M.G. Manoj, Richa Joshi

**Affiliations:** 1 Department of Nuclear Medicine, Command Hospital, Lucknow, Uttar Pradesh, India; 2 Department of Pathology, Command Hospital, Lucknow, Uttar Pradesh, India; 3 Department of Oncosurgery, Command Hospital, Lucknow, Uttar Pradesh, India

**Keywords:** ano-uro-genital melanoma, FDG PET-CT, mucosal melanoma, UK guidelines, urethral melanoma

## Abstract

Urethral melanomas are a rare subtype of noncutaneous melanomas. The disease has a tendency to have skip lesions and early metastases as compared with cutaneous melanomas. The role of fluorine-18 fluorodeoxyglucose ( ^18^ F-FDG) positron emission tomography computed tomography (PET-CT) is well established in cases of cutaneous melanomas and is recommended by the National Comprehensive Cancer Network (NCCN) for stage IIB to IV cancer. There are no established guidelines on the management of noncutaneous melanomas; however, a recently published United Kingdom national guideline aims to streamline the management of ano-uro-genital melanomas. The guideline describes a very limited role in the use of ^18^ F-FDG PET-CT in this case scenario. The tendency to skip lesions, early metastases, involvement of brain parenchyma, and finally the usage of anti-PD-1 medications are key areas where ^18^ F-FDG PET-CT has shown superiority over CT scan. With this case report, we aim to highlight the strength of ^18^ F-FDG PET-CT in the management of urethral melanomas, which can be extrapolated to other ano-uro-genital melanomas.

## Introduction

Melanomas are highly aggressive tumors originating from the pigmented melanocytes and are broadly classified into cutaneous melanomas (CM) and noncutaneous melanomas (NCM) because of the difference in origin, genetic mutations, and biological behavior. Urethral melanomas are a rare subtype of ΝCM. NCM as the cancer group represents a condition with highly unmet clinical need. These tumors have a tendency toward skip lesions and early metastases. The clinical need ranges from an accurate estimation of disease burden to metastatic workup and early identification of a treatment response.

Fluorine-18 fluorodeoxyglucose ( ^18^ F-FDG) positron emission tomography computed tomography (PET-CT) involves whole-body imaging and accurately identifies metastatic sites of melanoma and has been included in the National Comprehensive Cancer Network (NCCN) guidelines for CM in stage IIB to IV cancers. However, in NCM management a very limited role is present as per existing literature and guidelines. Τhis case report describes a case of urethral melanoma in a female patient with discussion on the recent United Kingdom guidelines recommendations for the management of urethral melanoma.

## Case Report

A 71-year-old woman with no known comorbidities presented with complaints of straining on passing urine for the last 6 months. Τhere was no associated history of fever, dysuria, or hematuria and neither did she complain of burning micturition or associated weight loss. General physical examination was essentially normal, but a per vaginum evaluation revealed a pigmented growth encasing the urethral orifice. Biopsy of the lesion confirmed the diagnosis of urethral melanoma.

**Fig. 1:**
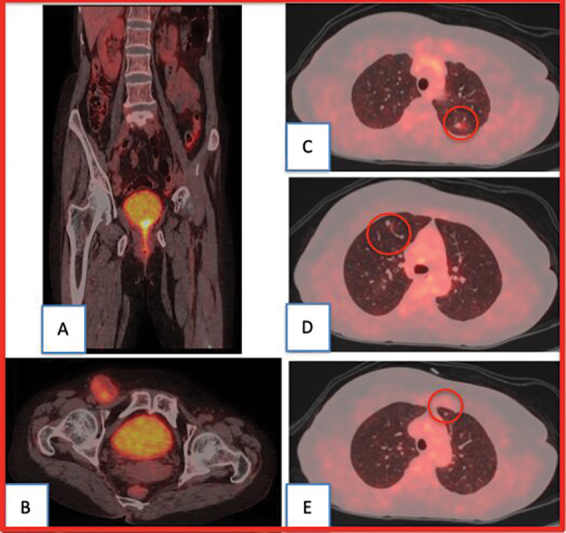
( **A** ) ^18^ F-FDG PET-CT coronal section image showing a metabolically active lesion involving the entire urethra with infiltration into the neck of the urinary bladder. ( **B** ) ^18^ F-FDG axial section at the level of the lower pelvic showing FDG avid right inguinal lymph node **.** ( **C** – **E** ) Axial section showing ^18^ F-FDG avid parenchymal nodules in bilateral lung fields.

An ^18^ F-FDG PET-CT scan was performed for a metastatic workup. The scan findings revealed metabolically active lesions involving the entire urethra with infiltration into the neck of the urinary bladder ( Fig. 1A ). Metastasis was noted in the form of FDG avid right inguinal lymph nodes along with FDG avid parenchymal nodules in bilateral (B/L) lung fields ( Fig. 1B–E ).

Based on the PET-CT findings of metastatic disease, the patient was counseled by the tumor board about disease severity and treatment intent. The patient underwent an anterior exenteration (AΕ) along with B/L inguinal lymph node dissection with ileal conduit diversion.

**Fig. 2:**
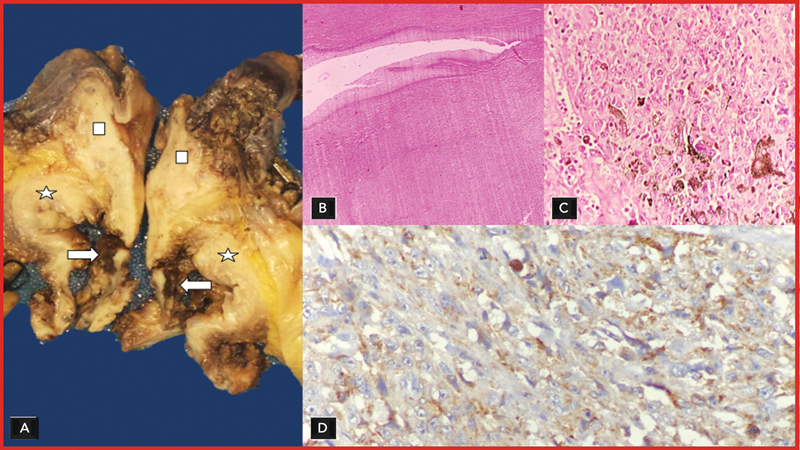
( **A** ) A gross specimen showing a pigmented urethral mass involving the entire length of the urethra. ( **B** , **C** ) Low- and high-power resolution vesicular nuclei and cytoplasmic melanin pigment. ( **D** ) The specimen showing immunohistochemical (IHC) positivity for HMB45 granular cytoplasmic expression.

Gross specimen evaluation revealed a pigmented urethral mass involving the entire length of the urethra ( Fig. 2A ). The high-power evaluation revealed vesicular nuclei and cytoplasmic melanin pigment and the immunohistochemistry (IHC) was positive for HMB45 granular cytoplasmic expression ( Fig. 2B–D ). The patient is presently under postsurgical rehabilitation and is being managed with PD-1 nivolumab.

## Discussion

Urethral melanoma is a rare type of NCM and comprises less than 1% of all melanomas. NCM differ from CM in their genetic profile and biological behavior showing higher incidence of c-KIT mutation and often present with distant metastases signifying poor prognosis. 1 2 3 In all, 55.4% of NCM originate from the mucosa of the upper aerodigestive tract, 18% from the female genital tract, 23.8% from the anorectal region, and only 2.8% of the mucosal melanomas originate from the urinary tract. 1 4

Urethral and bladder melanomas are subtypes of urinary tract melanomas. Distal urethra is the most common site of occurrence of urethral melanomas. 5 6 7 8 9 Women are affected more than men and the disease is commonly seen in women with white ethnic origin. 6 7 The disease affects the elderly usually above the age of 60 years. 10 The patients present with symptoms of hematuria, a mass protruding per vaginum or with symptoms of urinary tract obstruction. 11 The overall 3-year survival has been reported to be approximately 27% and most of the patients present in advanced stages with regional and distant metastases. 12 13

In our case, the patient affected was an elderly female with chief complaints of obstructive urinary symptoms. The patient did not complain of hematuria and even the routine urine examination did not reveal the presence of blood. Clinically, the patient had pigmented mass at the urethral meatus along with palpable nontender lymph nodes and on ^18^ F-FDG PET-CT metastasis to the lung nodules was noted.

The Levine staging system has been traditionally used for disease staging and classifies the disease into the following: stage A (confined to the submucosa), stage B (involving the corpus spongiosum), stage C (extending beyond the corpus spongiosum), and stage D (involving the inguinal lymph nodes). The United Kingdom national guidelines for ano-uro-genital melanomas include the urethral melanomas under vulvovaginal melanomas for females and under penile melanomas for male patients. This guideline recommends use of American Joint Committee on Cancer (AJCC) staging used for carcinomas for staging of NCM. 12 The guideline recommends the use of PET-CT only in the evaluation of low-volume brain metastasis before planning a radical surgery, and does not include PET-CT for disease staging. For disease staging, the guideline recommends the use of CT scan of the chest, abdomen, and pelvis.

Clinically our case was Levine stage D due to involvement of the B/L inguinal lymph nodes. AJCC stage was stage IV T3N3M1 based on PET-CT scan finding of the lung nodules. The scan was able to identify the extent of the primary lesion, outline the extent of lymph nodal and lung metastases, and also ruled out brain metastases. In this scenario, a single scan answered all the clinical dilemmas, removing the need for a second imaging modality and decreasing the radiation burden of the patient.

Due to the extreme rarity of the disease, there are no established guidelines on the clinical management. Wide resection surgery with lymph node dissection, followed by chemotherapy or immunotherapy remains the primary intervention in such cases. 9 14 Surgical options can vary from urethral resection to radical cystectomy to AΕ. Our patient was made aware of the disease severity and extent and offered radical surgery. AE along with B/L inguinal lymph node dissection and ileal conduit diversion was performed.

Immunotherapy has changed the way melanomas are managed and various studies show programmed cell death ligand 1 (PDL-1) expression in NCM. 15 Vulvar and vaginal melanomas have high expression of PD-1 and PDL-1. 16 These studies show the potential role immune checkpoint inhibitors can play in NCM. PD-1 activation by tumor cells causes T cell tolerance and inhibits the immune response toward cancer cells. PD-1 inhibitors such as nivolumab and pembrolizumab have been used with good results in the setting of metastatic melanoma. Trials are also being conducted using therapy for c-KIT mutations with Imatinib and has shown good results. 17

The United Kingdom guidelines recommend testing for BRAF and c-KIT mutations in ano-uro-genital melanomas. It recommends the use of PD-1 inhibitors as the first line of treatment and to consider BRAF + MEK inhibitors in selected few stage III and IV patients with positive BRAF mutation. The guideline presently does not recommend use of imatinib for patients with c-KIT mutations. Our patient was managed further with nivolumab, a PD-1 inhibitor after the postsurgical rehabilitation.

Our case showed the presence of urethral melanoma (UrM) involving the entire urethra and was seen infiltrating into the trigone of the bladder. In this case, PET-CT was able to clearly delineate the site of the primary and showed the presence of metastases to the inguinal lymph nodes and lung parenchyma. The scan was also able to rule out brain metastases. This shows the advantage of the use of FDG PET-CT for melanoma staging as against using CT scan as recommended in the United Kingdom guidelines.

Also, in the follow-up of patients, the guideline recommends using CT of the chest, abdomen, and pelvis at 6-month intervals for the initial 3 years after surgery and at 12-month intervals for the 4th and 5th year. 12 Imaging of the brain parenchyma is optional as most patients of melanomas present in advanced stages and are managed with PD-1 inhibitors. Interim ^18^ F-FDG PET-CT can be used for response evaluation of patients on immunotherapy with the PERCIMT (PET response evaluation criteria for immunotherapy) criteria. 18 This makes a stronger case for evaluation of the follow-up patients with PET-CT scan, as it not only helps in better identification of the response to immunotherapy and identifies recurrence earlier but also rules out recurrence of disease in brain parenchyma.

## Conclusion

Present guidelines on the management of NCM provide a very limited role of ^18^ F-FDG PET-CT in melanomas. The United Kingdom guidelines stay silent on use of PET-CT for disease staging and in the follow-up evaluation of melanoma patients. PET-CT being a whole-body scan shows high sensitivity for detecting metastatic melanoma lesions, rules out brain metastases, can assess disease response to PD-1 inhibitors, and provides a more holistic assessment of disease burden in a single scan during follow-up. Thus, it saves crucial time from disease estimation to treatment initiation and decreases the radiation burden of the patient.

The limited role of PET-CT in NCM is attributed probably to disease rarity and low availability of the modality worldwide rather than lack of clinical awareness. This results in fewer publications emphasizing on the role of FDG PET-CT in NCM. However, with improved availability, the scenario is going to change. We, at our center, evaluate all melanoma patients with an ^18^ F-FDG PET-CT because of its many advantages and recommend the same.
